# Papillary Fibroelastomas: 16-Year Single-Center Experience

**DOI:** 10.21470/1678-9741-2023-0431

**Published:** 2025-02-05

**Authors:** Strahinja Mrvic, Ranko Zdravkovic, Andrej Preveden, Novica Kalinic, Ilija Bjeljac, Aleksandar M Milosavljevic, Mirko Todic

**Affiliations:** 1 Clinic of Cardiovascular Surgery, Institute of Cardiovascular Diseases of Vojvodina, Sremska Kamenica, Serbia; 2 Faculty of Medicine, University of Banja Luka, Banja Luka, Bosnia and Herzegovina; 3 Faculty of Medicine, University of Novi Sad, Novi Sad, Serbia

**Keywords:** Heart Ventricules, Cardiac Papillary Fibroelastoma, Aortic Valve, Tricuspid Valve, Mitral Valve, Sudden Death, Echocardiography

## Abstract

**Introduction:**

Papillary fibroelastoma is a rare primary benign cardiac tumor. The majority of patients are asymptomatic. Complications may result in embolic events, syncope, ventricular arrhythmias, and sudden death. In this study, we report on a series of papillary fibroelastomas documented in our institution.

**Methods:**

Medical history records from period between 2007 and 2022 were reviewed for all the patients diagnosed with cardiac papillary fibroelastomas treated surgically and confirmed histologically. Clinical, tumor, and demographic characteristics, echocardiography findings, and treatment modalities were analyzed.

**Results:**

In a sixteen-year period, 12 cases of papillary fibroelastomas were documented. The percentage of female patients was 83.3%. The average age was 59.0 ± 11.2 years. Average diameter of tumor was 1.2 cm. The aortic valve was the most common origin site, with six cases (50%). In two cases (17%), the mitral valve was involved. There were single cases of tumor (8% each) found on the tricuspid valve, in the left atrium, in the left ventricle, and in the right ventricle. All patients were treated successfully by complete resection.

**Conclusion:**

PFEs are generally small and single tumors. Complete surgical resection of the tumor has a good prognosis and is a safe, efficient, and definitive treatment.

## INTRODUCTION

Primary cardiac tumors are rare, with an incidence rate of 0.0017%– 0.019% in the autopsy series^[1]^. In most cases, these are myxomas, while papillary fibroelastomas (PFEs) are in second place, with < 10% of the total number of primary heart tumors^[1]^. The majority of patients are asymptomatic. If symptomatic, they are most often presented with embolic complications, syncope, ventricular arrhythmias, and sudden death^[1,2]^. These tumors are typically located on the left side of the heart, while the right side localization is very rare^[3]^. In most cases, diagnosis is made by transthoracic echocardiography (TTE) or transoesophageal echocardiography (TEE). Diagnostic methods such as 3D echocardiography, cardiac magnetic resonance, and multi-slice spiral computed tomography could be used for more precise identification of the tumor^[4]^. Definitive diagnosis of PFE requires confirmation by histopathological analysis, since imaging presentation can often be misleading. Although there are no precise guidelines regarding management of PFE, surgical treatment is the preferred method of choice. Complete removal of the tumor without any fragmentation or residues must be ensured in order to achieve the optimal outcome^[5]^.

The aim of the present study was to analyze and present the characteristics and outcomes of PFE cases treated at our institution.

## METHODS

This is a retrospective observational study, in which we analyzed the data of patients with PFE, who underwent surgical treatment at the Institute of Cardiovascular Diseases of Vojvodina. The observation period was from 01 January 2007 until 31 December 2022. Patients’ data was collected from our hospital information system. The study analyzed demographic characteristics (patients’ age at the time of surgical intervention and sex), localization and the size of the tumor, clinical presentation, comorbidities, surgical techniques, and histopathological findings. The imaging method used for setting the diagnosis was TTE or TEE.

### Surgical Techniques

All the patients were operated in general endotracheal anesthesia, via total median sternotomy and with the use of cardiopulmonary bypass (CPB). In most cases (eight patients [67%]), isolated tumor excision was performed; in two cases (17%), tumor excision was performed with aortic valve replacement; in one (8%), it was performed with coronary artery bypass grafting (CABG); and in one case (8%), it was performed together with CABG and mitral valve repair. After tumor excision, intraoperative TEE was performed to confirm complete removal without sequelae

### Statistical Analysis

In this retrospective study, among the statistical methods, descriptive statistics measures were used: mean value, standard deviation, median, percentiles, and percentages.

## RESULTS

In a sixteen-year period, 12 cases of patients with PFE were documented. The average patients’ age was 59 ± 11.2 years, and 83% were female. Four patients (33%) were smokers. Six (50%) of the patients were asymptomatic, and those with symptoms presented with chest pain and fatigue. Four (33%) patients reported dyspnea and palpitations, three (25%) had a new-onset atrial fibrillation, and one (8%) patient had a transient ischemic attack (Table 1).

**Table 1:** The most common symptoms in a patient with papillary fibroelastoma.

Variable	No (%)
Chest pain	6 (50)
Fatigue	6 (50)
Dyspnea	4 (33)
Palpitations	4 (33)
Fainting	4 (33)
Atrial fibrillation	3 (25)
Transient ischemic attack	1 (8)

The most common associated diseases were arterial hypertension (nine patients), dyslipidemia (five patients), diabetes (three patients), chronic obstructive pulmonary disease (two patients), and atrial fibrillation (one patient) (Table 2).

**Table 2:** Common associated diseases in a patient with papillary fibroelastoma.

Variable	No (%)
Arterial hypertension	9 (75)
Dyslipidemia	5 (42)
Diabetes mellitus	3 (25)
Chronic obstructive pulmonary disease	2 (17)
Atrial fibrillation	1 (8)

The average left ventricular ejection fraction was 61.8 ± 7.2%. Average diameter of the tumor was 1.2 ± 0.5 cm. The average cardiac arrest time (on CPB) was 27 minutes, and the average total CPB time was 30.4 ± 18.7 minutes. The average number of days spent in the intensive care unit was 1.3 ± 0.5, and the average length of hospitalization was 7.9 ± 1.4 days. These characteristics are presented in Table 3.

**Table 3:** Cardiac and operative characteristics of patients with papillary fibroelastoma.

Variable	Mean ± SD
Ejection fraction (%)	61.8 ± 7.2
Dimension of tumor (cm)	1.2 ± 0.5
Cardiopulmonary bypass time (min.)	30.4 ± 18.7
ICU stay (days)	1.3 ± 0.5
Length of hospitalization (days)	7.9 ± 1.4
ICU=intensive care unit; SD=standard deviation	

The aortic valve was the most common site of origin (six patients). In two cases, the mitral valve was involved. One tumor was found on the tricuspid valve, in the left atrium, in the left ventricle, and in the right ventricle (Table 4).

**Table 4:** Tumor localization

Localization	No (%)
Aortic valve	6 (50)
Mitral valve	2 (17)
Tricuspid valve	1 (8)
Left atrium	1 (8)
Left ventricle	1 (8)
Right ventricle	1 (8)

## DISCUSSION

PFE are rare tumors. The precise etiology remains largely unknown. They originate mostly from the valvular endocardium. There are, however, several theories about the pathophysiology of PFE. One of the hypotheses is that these lesions are an acquired entity, and they begin as microthrombi. These microthrombi join in sites of minor endothelial damage. Over time, these microthrombi eventually evolve into PFE. These PFE continue to grow and can potentially embolize, causing resultant complications, including stroke, ventricular fibrillation, myocardial infarction, and sudden death^[6]^.

The incidence appears to be equal in males and females. But in our study, it was higher in females. It is predominantly found in patients aged between 40 and 80 years but many are diagnosed at approximately 60 years of age, which is consistent with our findings^[7,8]^.

In general, PFE may present in one of three ways: systemic — fever, arthralgia, weight loss, fatigue, and paraneoplastic syndromes —; cardiac — mass effect, arrhythmias, valve regurgitation, pericardial effusion with or without tamponade, dyspnea, chest discomfort, pre-syncope, or syncope—; and embolic — systemic or pulmonary thromboembolic phenomenon from the tumor—^[9]^. In our study, cardiac symptoms were the most common, while embolic neurological event was found in one patient.

PFE is often diagnosed incidentally with echocardiography, computed tomography, cardiac surgery, or at the autopsy. The imaging method of choice for PFE detection is echocardiography. TEE allows a more accurate assessment than TTE^[10,11,12]^. In the study by Tamin et al.^[4]^, both TTE and TEE revealed the tumor in 51% of patients, but in 33% of patients, the tumor could be detected only by TEE^[10,13]^. In all of our patients, the first presence of tumor was suspected solely on the basis of two-dimensional TTE, and after that confirmed with TEE.

When the presence of PFE is confirmed, the appropriate treatment choice — surgical or medical — is a matter of debate. Oral anticoagulation can be the treatment of choice if the tumor is not mobile. Most published studies agree on zero or very low in-hospital 30-day mortality in patients undergoing PFE excision^[14,15,16]^. We recommend surgical treatment for all patients with PFEs.

Across the literature, the most commonly involved valves are: aortic valve (35–63%), mitral valve (9–35%), tricuspid valve (6–15%), and, rarely, pulmonary valve (0.5–8%), which is consistent with our findings^[17,18]^.

They are usually solitary, slow growing benign tumors that are < 1.5 cm in diameter^[19,20,21,22,23]^. We obtained similar data in our study. PFE are often found downstream of the valve (aortic side of the aortic valve or ventricular side of the mitral valve)^[9,24,25]^. Rosic et al.^[1]^ reported a case with rather unique location of the tumor on the upstream side of the tricuspid valve.

### Limitations

Basically, the limitation of the study would be that this is a retrospective study with a small number of patients. However, considering that this is a rare diagnosis and that our institution has an average of less than one patient per year, we believe that it is still an important study, which answers some of the questions that are important for further studies.

## CONCLUSION

In this study, we present our institutional experience with PFEs. The diagnosis was most often made on the basis of echocardiography. Echocardiography, in first line TEE, provides the degree of structural resolution necessary to ascertain the location and the extent of anatomic and hemodynamic involvement. The definitive diagnosis is based on the characteristic histopathological features. Patients with PFE can be asymptomatic or symptomatic. However, due to the low operative mortality and the risk of embolization, we are of the opinion that all patients should undergo surgical excision of the tumor. Surgical removal of PFE is safe, efficacious, and definitive.

## Abbreviations, Acronyms & Symbols

CABG: Coronary artery bypass grafting
CPB: Cardiopulmonary bypass
ICU: Intensive care unit
PFE: Papillary fibroelastoma
SD: Standard deviation
TEE: Transoesophageal echocardiography
TTE: Transthoracic echocardiography

## References

[1] Rosic M, Zdravkovic R, Komazec N, Rosic V (2023). An unusual case of localization of papillary fibroelastoma on the
upstream side of the tricuspid valve. Kardiol Pol.

[2] Mehmood H, Charles C, Unai S, Maroo A (2020). Cardiac papillar y fibroelastoma originating from the coumadin
ridge and review of literature. BMJ Case Report.

[3] Rana Y, Tummala R, Engstrom K, Kukar N, Misra D (2021). Discovery of Tricuspid Fibroelastoma on
Echocardiography. Cureus.

[4] Šušak S, Okiljević B, Mrvić S, Rajić J, Zdravković R (2019). Surgical Treatment of Aortic Valve Fibroelastoma: A Case
Report. Scripta Medica.

[5] Zoltowska M, Sadic E, Becoats K, Ghetiya S, Azal Ali A, Sattiraju S (2021). Cardiac papillary fibroelastoma. Journal of Geriatric Cardiology.

[6] Devanabanda A, Lawrence S (2023). Lee. Papillary Fibroelastoma. StatPearls [Internet].

[7] Albuquerque L, Devens Trindade V (2011). Heart valve papillary fibroelastoma associated with cardioembolic
cerebral events. Rev Bras Cir Cardiovasc.

[8] Borsani P, Mariscalco G, Blanzola C, Leva C, Bruno VD, Cozzi G (2006). Asymptomatic cardiac papillary fibroelastoma: diagnostic
assessment and therapy. J Card Surg.

[9] Tyebally S, Chen D, Bhattacharyya S, Mughrabi A, Hussain Z, Manisty C, Westwood M, Ghosh AK, Guha A (2020). Cardiac Tumors: JACC CardioOncology State-of-the-Art
Review. JACC CardioOncol.

[10] Kolek M, Dvorackova J, Motyka O, Brat R (2020). Cardiac papillary fibroelastomas: A 10-year single-center
surgical experience and long-term echocardiographic follow-up
study. Biomed Pap Med Fac Univ Palacky Olomouc Czech Repub.

[11] Ngaage DL, Mullany CJ, Daly RC, Dearani JA, Edwards WD, Tazelaar HD (2005). Surgical treatment of cardiac papillary fibroelastoma: a single
center experience with eighty-eight patients. Ann Thorac Surg.

[12] Vandergoten P, Dendale P, Geukens R, Benit E, Vanbockrijk M, Mees U (1999). Right auricular fibroelastoma and paradoxical embolic
stroke. Acta Cardiol.

[13] Tamin SS, Maleszewski JJ, Scott CG, Khan SK, Edwards WD, Bruce CJ (2015). Prognostic and Bioepidemiologic Implications of Papillary
Fibroelastomas. J Am Coll Cardiol.

[14] Ikegami H, Andrei AC, Li Z, McCarthy PM, Malaisrie SC (2015). Papillary fibroelastoma of the aortic valve: analysis of 21
cases, including a presentation with cardiac arrest. Texas Heart Institute Journal.

[15] Dell'amore A, Albertini A, Lamarra M (2013). Twenty years experience in oncologic surgery for primary cardiac
tumors. G Chir.

[16] Mkalaluh S, Szczechowicz M, Torabi S, Dib B, Sabashnikov A, Mashhour A (2017). Surgery for Cardiac Papillary Fibroelastoma: A 12-Year Single
Institution Experience. Med Sci Monit Basic Res.

[17] Maurizio Parato V, Nocco S, Alunni G, Becherini F, Conti S, Cucchini U (2022). Imaging of Cardiac Masses: An Updated Overview. J Cardiovasc Echogr.

[18] Sun JP, Asher CR, Yang XS, Cheng GG, Scalia GM, Massed AG (2001). Clinical and echocardiographic characteristics of papillary
fibroelastomas: a retrospective and prospective study in 162
patients. Circulation.

[19] Boyacıoğlu K, Adnan Ak, Dönmez A, Çayhan B, Aksüt M, Altuğ Tunçer M (2018). Outcomes After Surgical Resection of Primary Non-Myxoma Cardiac
Tumors. Braz J Cardiovasc Surg.

[20] Thi Ngoc Chau A, Hien Nguyen Q, Nhat Pham H, Vo M, Ba Huynh D, Hai Nam Pham N (2022). Cardiac papillary fibroelastoma as a cause of acute coronary
syndrome. J Cardiol Cases.

[21] Zoltowska E, Sadic K, Becoats S, Ghetiya A.A, Ali S, Sattiraju S (2021). Cardiac papillary fibroelastoma. J Geriatr Cardiol.

[22] Jha K, Khouri M, Murphy D.M, Salustri A, Khan J.A, Saleh M.A, Von Canal F (2010). Papillary fibroelastoma of the aortic valve–a case report and
literature review. J Cardiothorac Surg.

[23] Mariscalco G, Bruno V.D, Borsani P, Dominici C, Sala A (2010). Papillary fibroelastoma: insight to a primary cardiac valve
tumor. J Card Surg.

[24] Capotosto L, Elena G, Massoni F, De Sio S, Carnevale A, Ricci S (2016). Cardiac Tumors: Echocardiographic Diagnosis and Forensic
Correlations. Am J Forensic Med Pathol.

[25] Rudienė V, Glaveckaitė S, Matačiūnas M (2022). Papillary fibroelastoma of the aortic valve. Kardiol Pol.

